# Metabolic alterations provide insights into *Stylosanthes* roots responding to phosphorus deficiency

**DOI:** 10.1186/s12870-020-2283-z

**Published:** 2020-02-22

**Authors:** Jiajia Luo, Yunxi Liu, Huikai Zhang, Jinpeng Wang, Zhijian Chen, Lijuan Luo, Guodao Liu, Pandao Liu

**Affiliations:** College of Tropical Crops, Hainan University, Institute of Tropical Crop Genetic Resources, Chinese Academy of Tropical Agriculture Sciences, Haikou, 570228 China

**Keywords:** Metabolome, *Stylosanthes*, Phosphorus deficiency, Root morphology, Flavonoid, Expansin

## Abstract

**Background:**

Phosphorus (P) deficiency is one of the major constraints limiting plant growth, especially in acid soils. *Stylosanthes* (stylo) is a pioneer tropical legume with excellent adaptability to low P stress, but its underlying mechanisms remain largely unknown.

**Results:**

In this study, the physiological, molecular and metabolic changes in stylo responding to phosphate (Pi) starvation were investigated. Under low P condition, the growth of stylo root was enhanced, which was attributed to the up-regulation of *expansin* genes participating in root growth. Metabolic profiling analysis showed that a total of 256 metabolites with differential accumulations were identified in stylo roots response to P deficiency, which mainly included flavonoids, sugars, nucleotides, amino acids, phenylpropanoids and phenylamides. P deficiency led to significant reduction in the accumulation of phosphorylated metabolites (e.g., P-containing sugars, nucleotides and cholines), suggesting that internal P utilization was enhanced in stylo roots subjected to low P stress. However, flavonoid metabolites, such as kaempferol, daidzein and their glycoside derivatives, were increased in P-deficient stylo roots. Furthermore, the qRT-PCR analysis showed that a set of genes involved in flavonoids synthesis were found to be up-regulated by Pi starvation in stylo roots. In addition, the abundances of phenolic acids and phenylamides were significantly increased in stylo roots during P deficiency. The increased accumulation of the metabolites in stylo roots, such as flavonoids, phenolic acids and phenylamides, might facilitate P solubilization and cooperate with beneficial microorganisms in rhizosphere, and thus contributing to P acquisition and utilization in stylo.

**Conclusions:**

These results suggest that stylo plants cope with P deficiency by modulating root morphology, scavenging internal Pi from phosphorylated metabolites and increasing accumulation of flavonoids, phenolic acids and phenylamides. This study provides valuable insights into the complex responses and adaptive mechanisms of stylo roots to P deficiency.

## Background

Phosphorus (P) is one of the essential macronutrients for plant growth. It involves in photosynthesis, energy conversion, signal transduction and biomacromolecule biosynthesis [1]. Soluble inorganic phosphate (Pi) is the major form of P that can be directly utilized by plants from soils [2]. However, P is easily fixed by organic compounds, iron (Fe) or aluminum (Al) oxides in soils [3]. Low Pi availability is a major constraint limiting crop growth and yield [4]. In modern agriculture, Pi-fertilizers are excessively applied to alleviate P deficiency [5]. Excess application of Pi fertilizers is adverse to environment-friendly agriculture [6]. Therefore, in order to maintain the sustainable development of agriculture, it is great important for understanding of how plants adaptation to Pi starvation, which will aid in breeding crop cultivars with high P efficiency [7].

Plants have evolved a series of adaptive mechanisms to enhance P acquisition efficiency (PAE) and P utilization efficiency (PUE) in Pi-limited soils [8]. The major adaptive strategies of plant increasing PAE include modification of root architecture and morphology [9], exudation of organic acids from root to rhizosphere [10], secretion of phosphatases and ribonucleases [11], enhancing the expression of Pi transporter [12] and symbiosis with arbuscular mycorrhiza (AM) or other plant growth-promoting rhizobacteria (PGPR) [13]. The dominant PUE-related strategies include replacement of membrane phospholipids by galactolipids and sulfolipids [14], remobilization Pi from the vacuolar P storage [15], and alternative metabolic bypass reactions, for example inorganic pyrophosphate (PPi) instead of Pi [16]. These complex responses can be achieved by the coordination of an elaborate P signaling network [17, 18]. Over the past few decades, several key regulators involved in this network have been functionally characterized, such as PHR1 (phosphate starvation response 1), SPX domain-containing proteins, PHO2 (a ubiquitin-conjugating E2 enzyme) and microRNA399 [19–21].

Low Pi availability induces dramatic changes in gene and protein expressions in plants [22, 23]. Transcriptomic and proteomic techniques have been widely used to investigate Pi starvation response (PSR) genes and proteins in various plants, such as *Arabidopsis thaliana* [24], rice (*Oryza sativa*) [25, 26], maize (*Zea mays*) [27–29] and masson pine *(Pinus massoniana*) [30, 31]. A group of PSR genes and proteins have been implicated in P uptake and reallocation, P-containing metabolites catabolism, organic acid metabolism, signal regulating, and cell wall modification [32]. For example, expansins are superfamily proteins that modify cell wall loosening, leading to cell extension [33]. *GmEXLB1*, one of Pi starvation-inducible expansin genes, identified by transcriptomic analysis in soybean (*Glycine max*), has been characterized to be involved in P acquisition by regulating root growth [34]. Recently, a pair of rice vacuolar Pi efflux transporters (OsVPE1 and OsVPE2) identified by vacuolar membrane proteomics, have been demonstrated to participate in vacuole Pi remobilization [35]. Although the molecular properties of several PSR genes and proteins have been demonstrated, biological functions of numerous PSR genes and proteins have not been well documented.

In addition to the changes in gene and protein abundances, metabolic alterations are observed in P-deficient plants. Metabolomic analysis using gas or liquid chromatography mass spectrometry (GC-MS or LC-MS) technique is a useful tool in investigating metabolism mechanic response of plants to Pi starvation [36]. Metabolite profiling analyses have been carried out in several plants response to P deficiency, such as Arabidopsis [37], rice [38], maize [39], barley (*Hordeum vulgare*) [40], wheat (*Triticum aestivum*) [41], soybean [42] and tea (*Camellia sinensis*) [43]. Furthermore, a set of metabolites differentially regulated by Pi starvation are conserved in different plants. For example, decreases in the levels of phosphorylated sugars are generally observed in P-deficient plants. However, significant variations in the changes of several amino acids abundances have been reported among different plant species. Furthermore, flavonoids belonging to the large family of phenylpropanoid metabolites, are implicated in plant growth, development and plant-environment interactions [44]. The effects of P deficiency on the levels of flavonoids have been analyzed via metabolomic analysis only in a few plant species, including Arabidopsis [37], soybean [42] and tea [43]. However, thousands of flavonoids have been identified in various plant species [44]. The responses of diverse flavonoids to low P stress are still unclear.

Stylo (*Stylosanthes* spp.) is an important forage legume that is widely used in agricultural systems in tropical and subtropical areas, where acid soils are widely distributed [45]. P deficiency, along with Al and manganese (Mn) toxicity, are considered as the major limiting factors for crop growth and production in acid soils [46]. Therefore, it is reasonable to believe that stylo is a pioneer tropical legume with extensive adaptation to acid soil-based abiotic stresses, especially for P deficiency. Recently, transcriptome and proteome profiles have been used to analyze the responses of stylo to Al and Mn toxicity [47–49]. However, metabolic alterations of stylo roots in response to P deficiency have yet to be reported. In this study, changes in growth performance, PSR genes expressions and metabolome profiling in response to Pi starvation were investigated in stylo roots.

## Results

### Physiological responses of stylo to Pi starvation

**Fig. 1:**
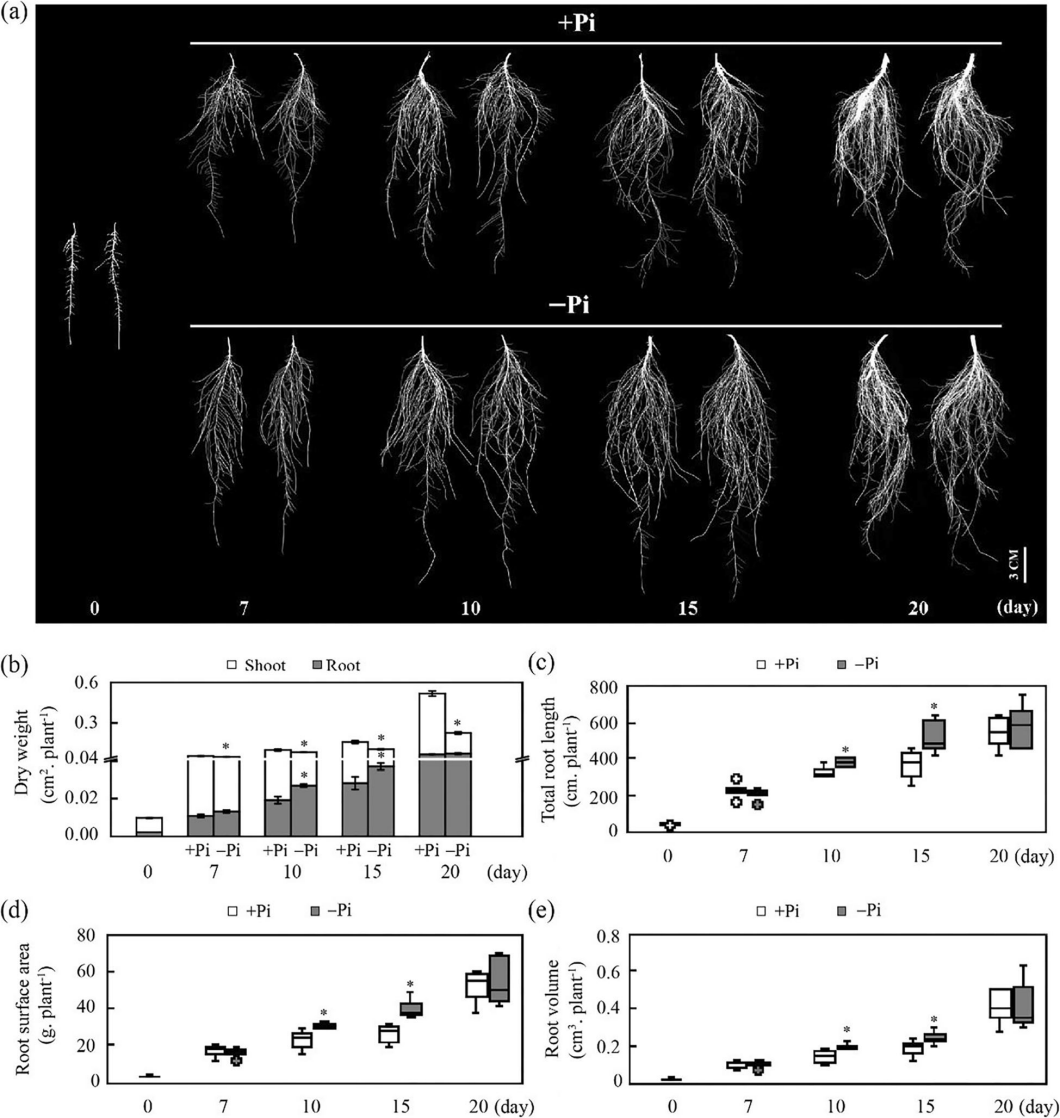
Effects of Pi availability on stylo growth. **a** Root growth performance; **b** dry weight of shoots and roots; **c** total root length; **d** root surface area; **e** root volume. Uniform 7d-old stylo seedlings were transferred into nutrient solution with (+Pi) or without (-Pi) 250 μmol/L KH_2_PO_4_ additions for 0, 7, 10, 15 or 20 d. Quartile (box), maximum and minimum (whiskers) and outlying values (circles) are shown in boxplots. Each experiment included six biological replicates (*n* = 6). Error bars indicate standard error (SE). Asterisks represent significant differences between +Pi and -Pi treatments (*P* < 0.05). Bar = 3 cm

To assess the dynamic alternations of stylo under P-sufficient (+Pi) and P-deficient (-Pi) conditions, a time-course of hydroponic experiment was performed. Results showed that the decreased in the shoot dry weight was observed after 7 d of -Pi treatment, and the differences between -Pi and +Pi became increasing significantly as the treatment time increased (Fig. 1). Although no difference in root dry weight was found under two P treatments for 7 d. Root dry weight was increased by 40.3 and 31.2% after 10 and 15 d of -Pi treatment compared to that in +Pi treatment, respectively (Fig. 1a, b; Additional file 1: Figure S1). Consistently, the total root length, root surface area and root volume were increased after 10 and 15 d of -Pi treatment (Fig. 1c, d, e). The maximum increased ratios of total root length and root surface area were 44.6 and 56.1% at 15 d of P treatments, respectively (Additional file 1: Figure S2a, b).

The ratio of root/shoot was significantly increased under Pi starvation. Furthermore, the maximum ratio of root/shoot was reached at 15 d of -Pi treatment, which was 2.6-fold higher than that of +Pi treatment (Additional file 1: Figure S3). After 15 d of -Pi treatment, although total P content was declined, the activity of root acid phosphatase (APase) and phenylalanine ammonia (PAL) were increased by 209.0 and 100.5%, respectively (Additional file 1: Figure S4a, b, c). Similarly, root total phenol, flavonoid, and total antioxidant capacity (T-AOC) were also increased by -Pi treatment compared to those of +Pi treatment (Additional file 1: Figure S4d, e, f). Furthermore, it was found that the internal malate concentration of stylo roots was decreased by 69.5%, but the external malate exudation rate of stylo roots was increased by 44.3% under -Pi treatment (Additional file 1: Figure S4g, h). These results suggest that a series of morphological and physiological changes occur in stylo in response to Pi starvation.

### Characterization of *expansin* gene family in stylo roots

**Fig. 2:**
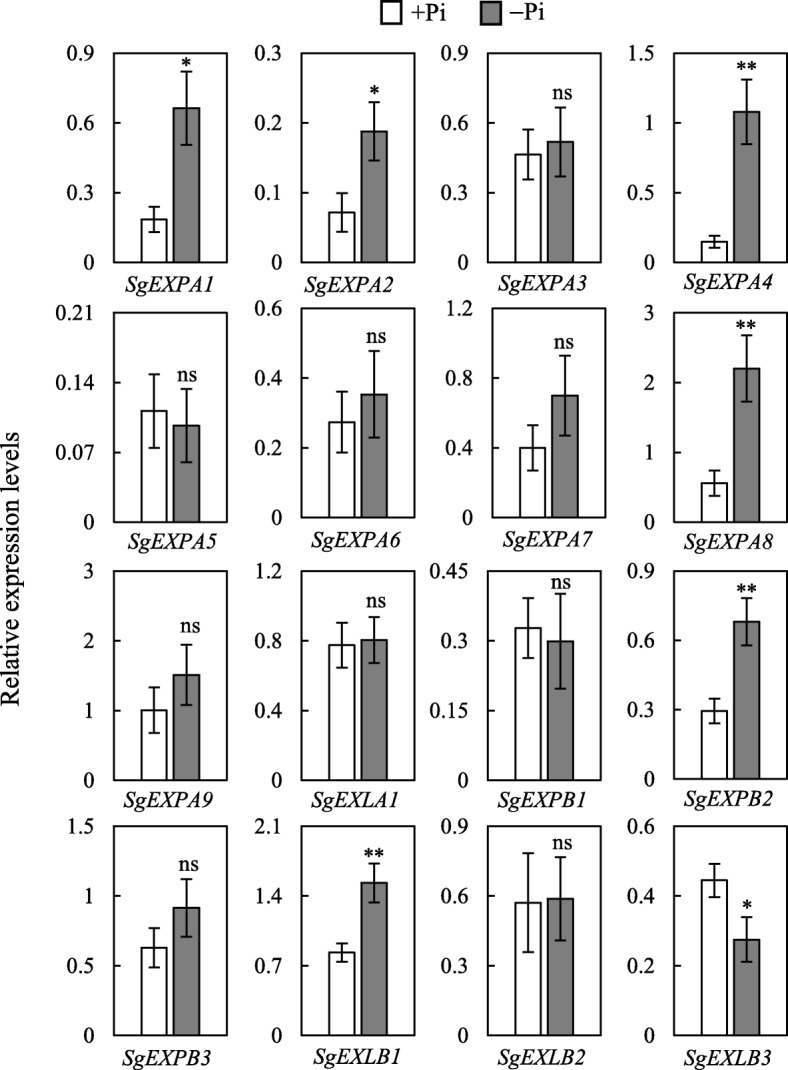
Expression analysis of genes encoding expansin proteins at two P levels. Stylo seedlings were exposed to +Pi (250 μmol/L KH_2_PO_4_) and -Pi (0 μmol/L KH_2_PO_4_) treatments for 15 d. The roots were harvested for qRT-PCR analysis. Each bar represents the mean of four replicates with SE (*n* = 4). Asterisks indicate significant differences between +Pi and -Pi treatments in the Student’s *t*-test (*: *P* < 0.05, **: 0.001 < *P* < 0.01, ***: *P* < 0.001), ns indicates no significant difference

Expansin protein family, localized to the cell wall, is implicated in regulating plant root growth [50]. In this study, a total of 16 *expansin* genes were identified in stylo (Additional file 2: Table S1). Phylogenetic analysis showed that 16 stylo *expansin* members were divided into four subfamilies, including 9 *SgEXPA*, 1 *SgEXLA*, 3 *SgEXPB* and 3 *SgEXLB* (Additional file 1: Figure S5). The expression patterns of these genes were further analyzed in roots of stylo with 15 d of P treatments. Results showed that 4 *SgEXPAs* (*SgEXPA1*, *2*, *4* and *8*), *SgEXPB2* and *SgEXLB1* were up-regulated, whereas *SgEXLB3* was down-regulated by Pi starvation. The expressions of the remaining stylo *expansin* genes were not affected by low P treatment (Fig. 2). These results suggest that some of the stylo *expansin* genes participate in modifying root growth under low P condition.

### Overview of metabolome in stylo roots response to Pi starvation

**Fig. 3:**
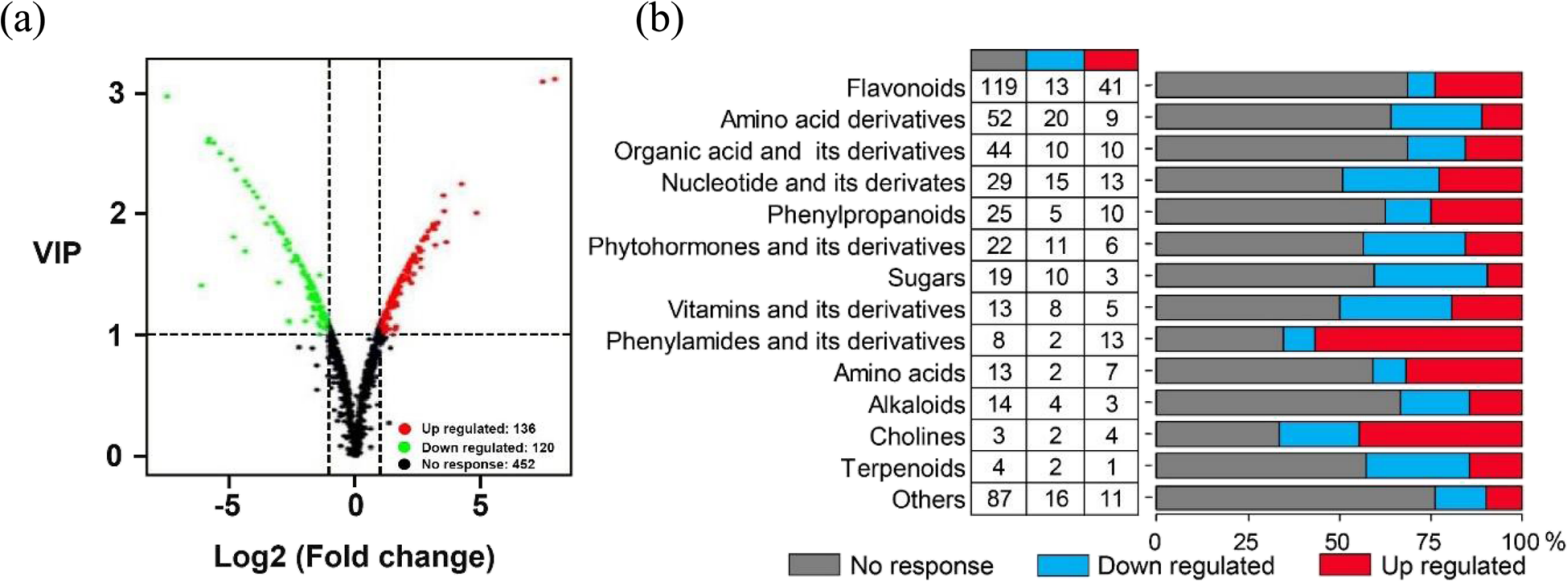
Overview of metabolomic changes in stylo roots at two P treatments. **a** The volcano diagram of distribution metabolites in stylo roots response to P deficiency. Red circles, green circles and black circles indicate up regulated, down regulated and no response metabolites, respectively. **b** Category of differentially accumulated metabolites (DAMs) in stylo roots during low P stress. Data are shown as the number of metabolites, DAMs are defined by conforming the variable importance in project (VIP) ≥ 1.0 and -Pi/+Pi ≥2.0 or ≤ 0.5. Up-regulated: -Pi/+Pi ≥2.0, down-regulated: -Pi/+Pi ≤0.5

To evaluate metabolome response to Pi starvation, an LC-MS/MS analysis was performed on stylo roots under two P treatments. A total of 708 metabolites were identified in stylo roots at two P levels (Additional file 3: Table S2). Principal component analysis (PCA) showed that principal component one (PC1) nicely defined the difference between +Pi (triangles) and -Pi (circles) stylo materials, which represented about 80.15% of the variation (Additional file 1: Figure S6a). The metabolites with the ratio of -Pi/+Pi more than 2 or less than 0.5 and the variable importance in project (VIP) more than 1.0 were considered as differentially accumulated metabolites (DAMs). A total of 256 DAMs were identified in stylo roots at two P treatments, including 136 low P up-regulated metabolites and 120 low P down-regulated metabolites (Fig. 3a), which can be clustered into the up-regulation cluster (color in red) and down-regulation cluster (color in blue), respectively (Additional file 1: Figure S6b). The root samples were also clustered into +Pi and -Pi treatment branches (Additional file 1: Figure S6b). All of the identified 256 DAMs were classified into 14 categories, including flavonoids, phenylpropanoids, phenylamides and its derivatives, amino acids and cholines, the numbers of the up-regulated these DAMs were higher than those of the down-regulated DAMs (Fig. 3b). However, the numbers of up-regulated amino acid derivatives and sugars were less than those of the down-regulated (Fig. 3b).

### Changes in sugars, cholines, nucleotide and its derivatives in stylo roots response to Pi starvation

**Fig. 4:**
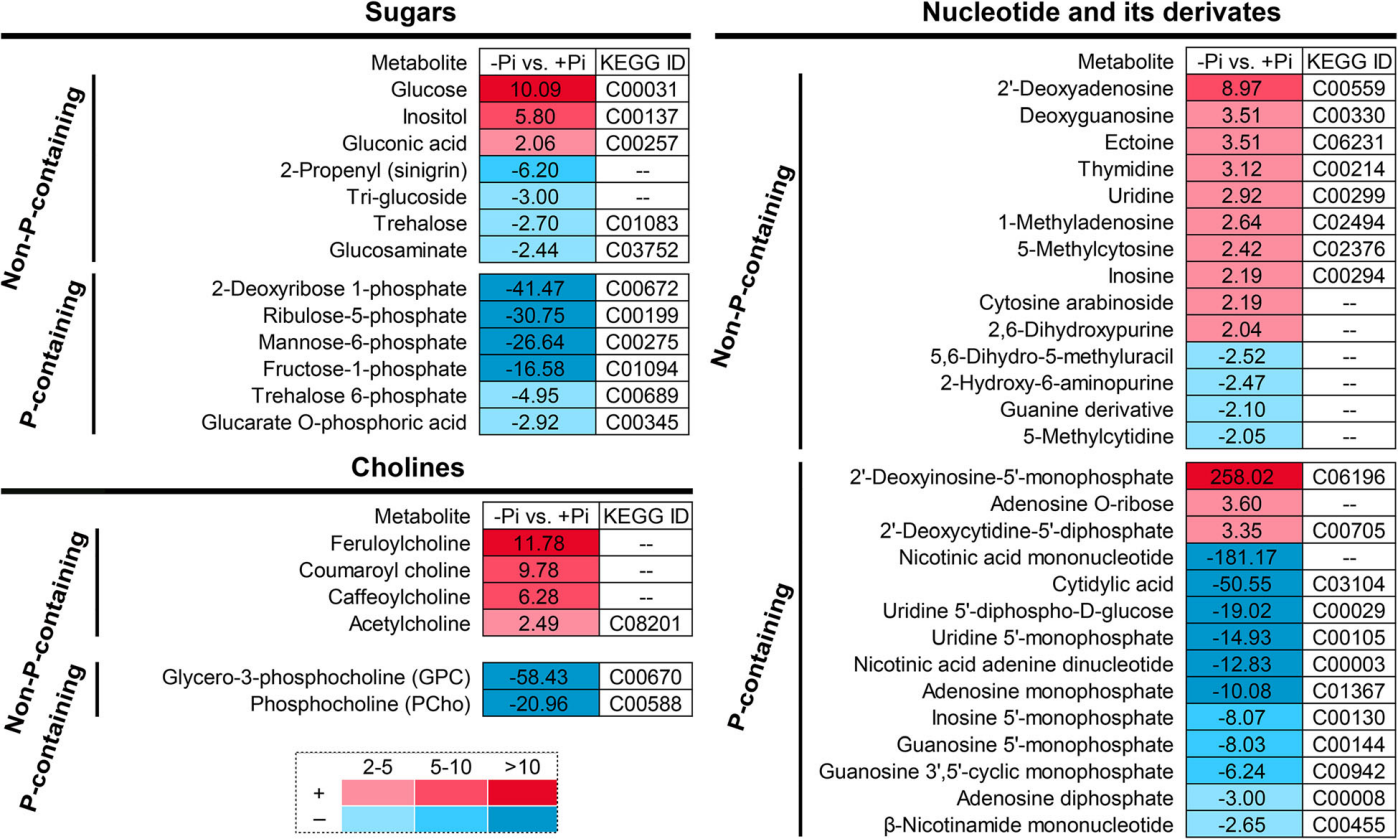
Effects of Pi availability on the relative levels of sugars, cholines, nucleotide and its derivatives in stylo roots. Data are shown as heatmap for the response ratio of -Pi (0 μmol/L KH_2_PO_4_) and +Pi (250 μmol/L KH_2_PO_4_) (*n* = 3). Response ratios are shown as positive numbers for an increase or negative inverted number for a decrease. In addition, the Kyoto Encyclopedia of Genes and Genomes identity (KEGG ID) are obtained from KEGG database (https://www.kegg.jp/). “--” in this figure represents the metabolite without an ID in KEGG database

Based on the existence of phosphate group in metabolites, sugars, cholines, nucleotide and its derivates can be classified into two groups, namely P-containing or non-P-containing metabolites. For the 13 sugars-related DAMs, the levels of 6 P-containing sugars were declined by-Pi treatment. Among them, 2-deoxyribose 1-phosphate, ribulose-5-phosphate, mannose-6-phosphate and fructose-1-phosphate were decreased by more than 10-fold under -Pi treatment compared to those under +Pi treatment. However, the levels of 3 non-P-containing sugars were significantly increased, including glucose, inositol and gluconic acid (Fig. 4). Furthermore, the levels of 4 non-P-containing cholines were increased, but the levels of P-containing cholines, such as glycerol-3-phosphocholine (GPC) and phosphocholine (PCho), were decreased by 58.43- and 20.96-fold under -Pi treatment compared to those under +Pi treatment, respectively (Fig. 4).

In addition, 28 DAMs were identified as nucleotide and its derivates. Among them, the levels of 10 out of 14 non-P-containing nucleotides were increased, while the levels of 11 out of 14 P-containing nucleotides were decreased in stylo roots exposed to low P stress. Nicotinic acid mononucleotide, cytidylic acid, uridine 5′-diphospho-D-glucose, uridine 5′-monophosphate, nicotinic acid adenine dinucleotide and adenosine monophosphate were declined by more than 10-fold in P-deficient stylo roots (Fig. 4).

To detect the response of hydrolases participating in P-containing metabolites catabolism to low P stress, the expressions of 5 stylo purple acid phosphatase (*PAP*) and 3 ribonuclease (*RNS*) genes were analyzed by quantitative real-time polymerase chain reaction (qRT-PCR). Result showed that the expressions of 4 out of 5 *SgPAPs* were up-regulated by-Pi treatment, especially for *SgPAP10*/*12*/*23*, and the expressions of 3 out of 4 *SgRNSs* were enhanced under low Pi stress (Additional file 1: Figure S7).

### Alterations of amino acid and its derivatives in stylo roots response to P deficiency

**Fig. 5:**
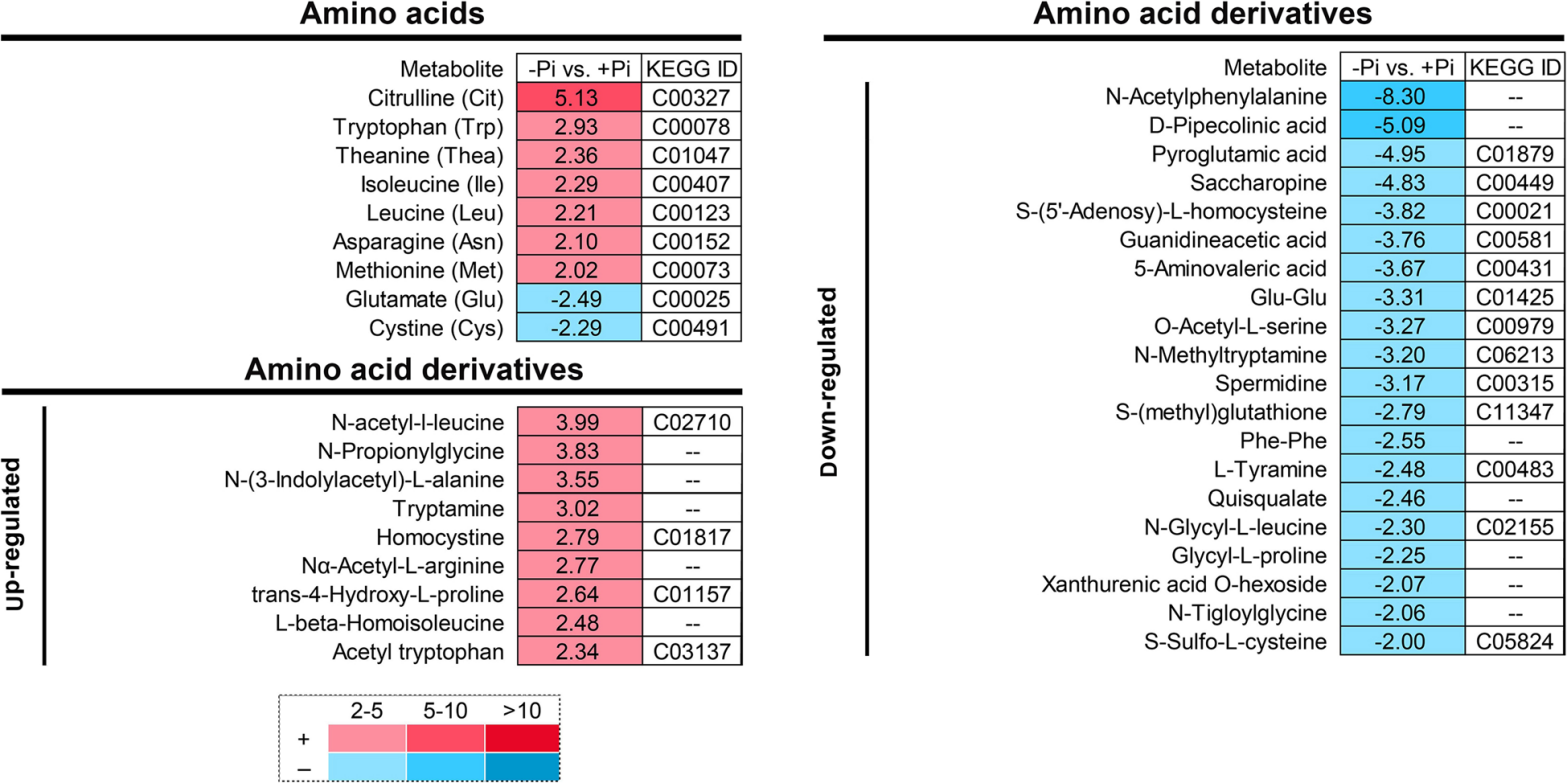
Alternations in amino acid and its derivatives in stylo roots response to Pi starvation. Data indicate the response ratio of -Pi (0 μmol/L KH_2_PO_4_) and +Pi (250 μmol/L KH_2_PO_4_) (*n* = 3). Positive numbers and negative inverted numbers represent an increase and decrease in response to P deficiency, respectively. KEGG IDs are obtained from KEGG database (https://www.kegg.jp/). “--” in this figure represents the metabolite without an ID in KEGG database

Among the identified amino acids, 9 out of 22 were considered as DAMs (Additional file 4: Table S3), 7 out of 9 amino acids-related DAMs were significantly induced under -Pi treatment, whereas glutamate (Glu) and cystine (Cys) were decreased by Pi starvation in stylo roots. Citrulline was the amino acid with the highest level (more than 5-fold) (Fig. 5). In addition, 81 amino acid derivatives were also identified in this study. Among them, 29 amino acid derivatives were DAMs, including 9 up-regulated and 20 down-regulated amino acid derivatives (Additional file 4: Table S3; Fig. 5).

### Analysis of flavonoids in stylo roots response to P deficiency

A total of 54 DAMs belonged to flavonoids, including 23 flavones, 16 flavonols, 6 flavanones, 4 isoflavones, 2 flavanols, 2 anthocyanins and 1 chalcone. The accumulation of a larger number of flavonoids was increased in stylo roots under low P stress (Additional file 1: Figure S8).

**Fig. 6:**
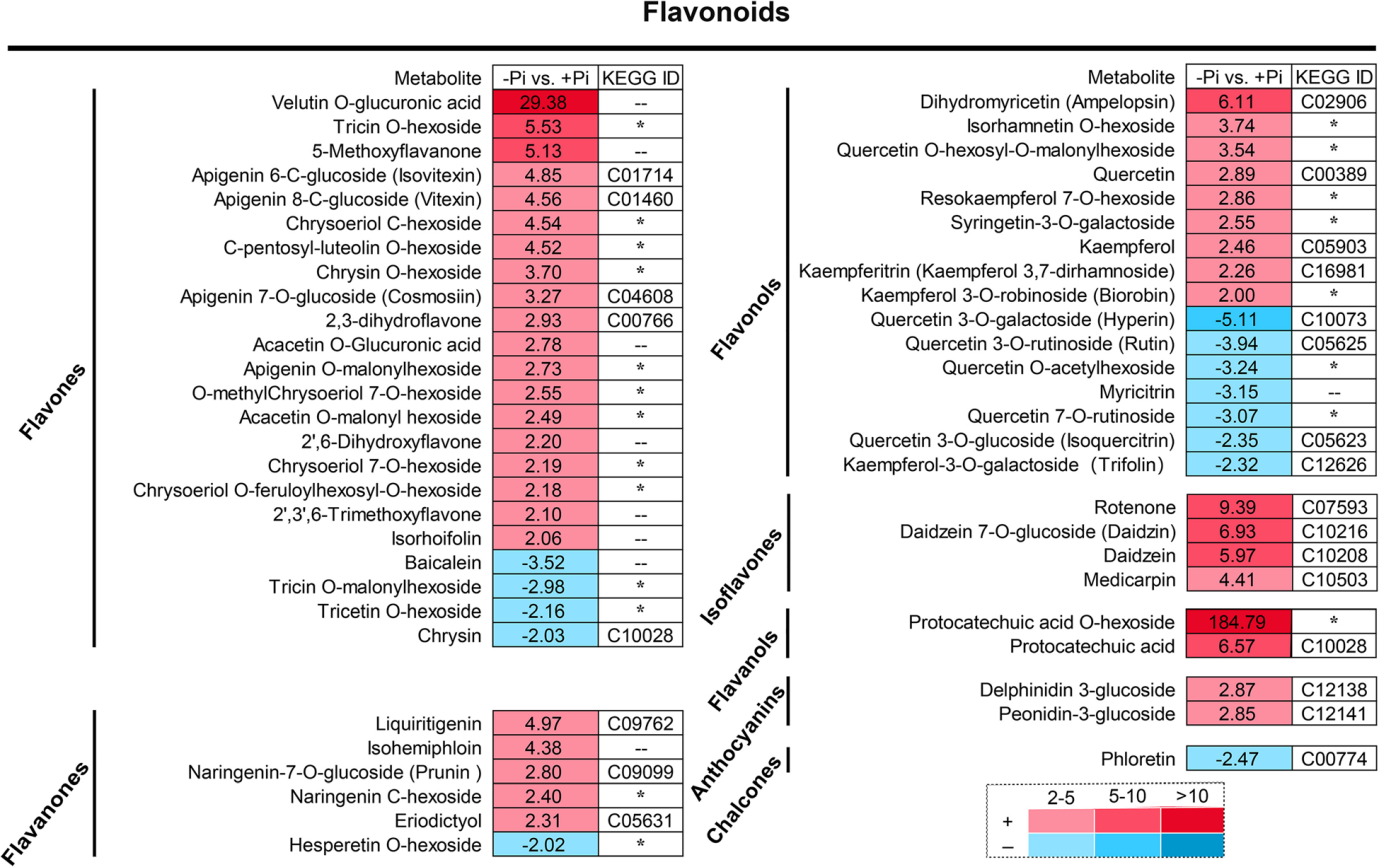
Analysis of flavonoid metabolites in stylo roots at low P stress. Fold changes of metabolites (-Pi/+Pi) are shown as numbers, and the ratio of -Pi/+Pi less than 0.5 is inverted as negative a number (*n* = 3). The metabolite ID of KEGG database is listed in the last column, “--” indicates the metabolite without an ID in KEGG database, “*” represents the metabolite without an ID in KEGG database and belonging to a glycoside derivative

**Fig. 7:**
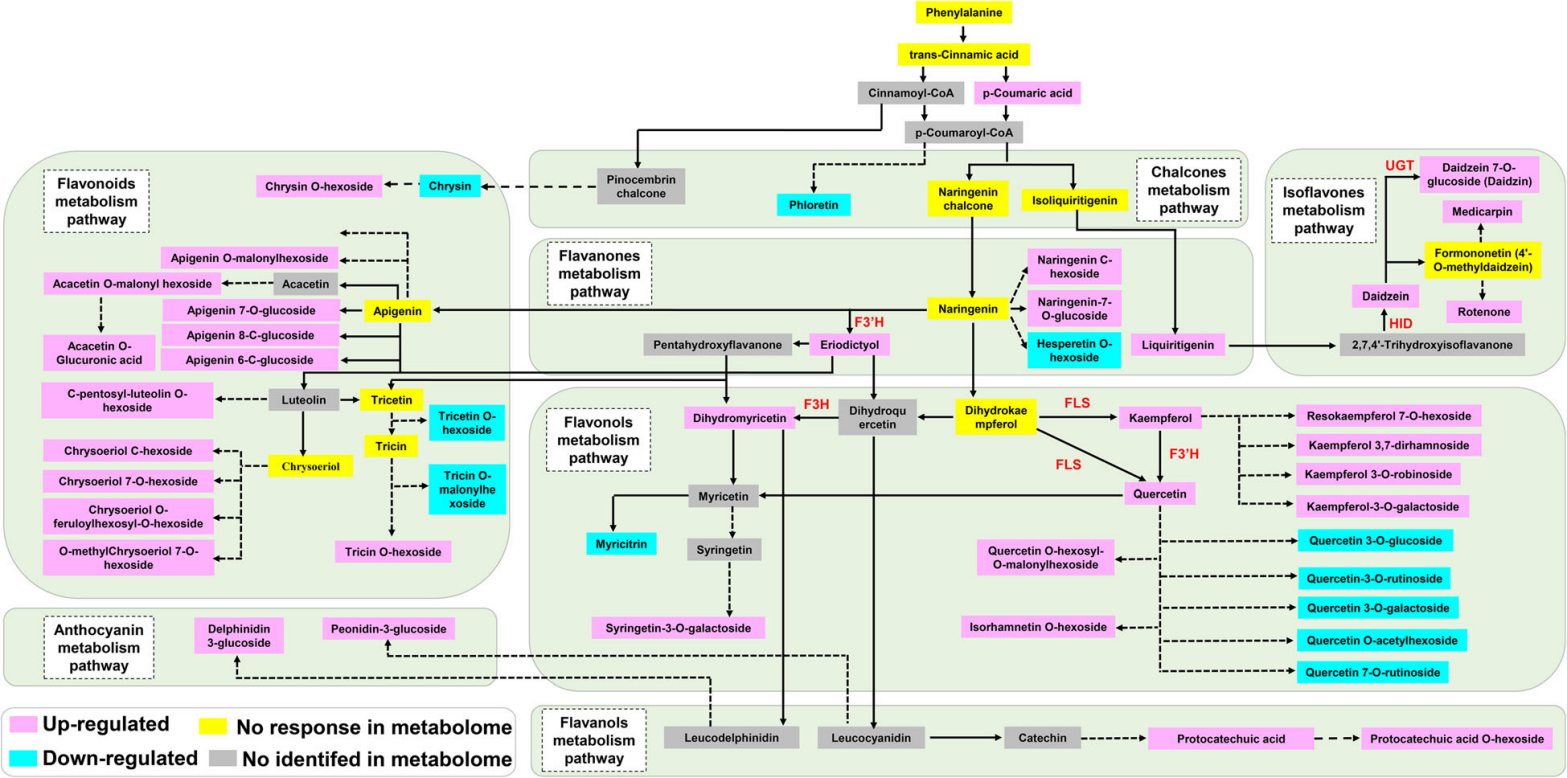
Flavonoid metabolism pathway in stylo roots response to P deficiency. The pathway was designed based on KEGG pathways (http://www.kegg.jp/kegg/pathway.html) as described by Zhang et al. [51] and Kc et al. [43]. The light red, blue, yellow and grey colors indicate up-regulated, down-regulated, no response and no identified metabolites in metabolome, respectively. The solid arrows show direct and dotted arrows represent indirect and speculated steps in the pathway. Important genes are marked on the metabolic procedure

For the differentially accumulated flavones, the accumulation of 19 out of 23 flavones was significantly increased, including 4 glycoside derivatives of apigenin and 4 glycoside derivatives of chrysoeriol, while the concentrations of the remaining flavones were declined by -Pi treatment (Fig. 6; Fig. 7).

For the differentially accumulated flavonols, the levels of 9 out of 16 flavonols was increased, whereas the levels of the other 7 flavonols were decreased by -Pi stress. Although the levels of kaempferol and quercetin were increased under Pi starvation, their glycoside derivatives displayed different responses to low P stress. Three glycoside derivatives of kaempferol were up-regulated, whereas 5 glycoside derivatives of quercetin were down-regulated by low P stress (Fig. 6; Fig. 7).

For the differentially accumulated flavanones, the abundances of the most flavanones were increased in stylo roots under low P stress, except hesperetin O-hexoside. The liquiritigenin was found to be the strongest up-regulated by 4.97-fold under P deficiency (Fig. 6; Fig. 7). Similarly, the concentrations of 4 isoflavones were increased under -Pi stress. The concentrations of three isoflavones, including rotenone, daidzein and daidzein 7-O-glucoside, were increased by more than 5-fold in low P-deficient roots (Fig. 6).

**Fig. 8:**
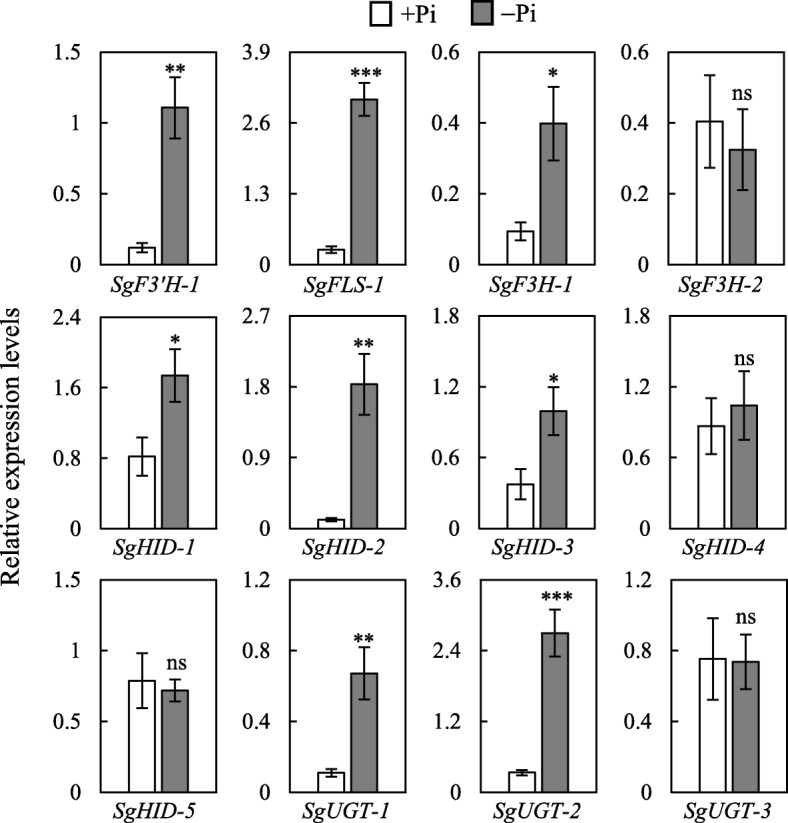
Expression analysis of candidate key genes associated with flavonoid metabolism in stylo roots at low P stress. The expression of 12 genes were analyzed in roots of stylo at +Pi and -Pi treatments for 15 d. Transcript expression levels were normalized using housekeeping gene *SgEF1α*. Each bar represents the mean of four replicates with SE (*n* = 4). Asterisks indicate significant differences between +Pi and -Pi treatments in the Student’s *t*-test (*: *P* < 0.05, **: 0.001 < *P* < 0.01, ***: *P* < 0.001), ns indicates no significant difference

Subsequently, the expressions of *2-hydroxyisoflavanone dehydratase* (*SgHID*) and *uridine diphosphate glycosyltransferase* (*SgUGT*) involved in daidzein and daidzein 7-O-glucoside synthesis, respectively, were further analyzed. qRT-PCR analysis showed that the expressions of 3 out of 5 *SgHIDs* and 2 out of 3 *SgUGTs* were up-regulated in stylo roots by low P stress (Fig. 8). In addition, the expressions of *flavonoid 3′-hydroxylase* (*SgF3’H*), *flavonol synthase* (*SgFLS*) and *flavanone 3-hydroxylase* (*SgF3H*) associated with flavonoids synthesis were also detected. Results showed that the expressions of *SgF3’H-1*, *SgFLS-1* and *SgF3H-1* were up-regulated in stylo roots under P deficiency (Fig. 8).

### Analysis of phenylpropanoids, phenylamides and its derivatives in stylo roots response to P deficiency

In addition to flavonoids, other differentially accumulated phenylpropanoids were also identified in this study, including 7 phenolic acids and 8 non-phenolic acid phenylpropanoids. For phenolic acids, the concentrations of 5 DAMs were significantly increased, especially for, caftaric acid, which was increased by more than 10-fold under -Pi treatment. For non-phenolic acid phenylpropanoids, the concentrations of 5 DAMs were enhanced, whereas 3 DAMs were decreased in roots exposed to low P treatment. Among the increased DAMs, the relative level of sesamin was increased by more than 10-fold in P-deficient stylo roots.

**Fig. 9:**
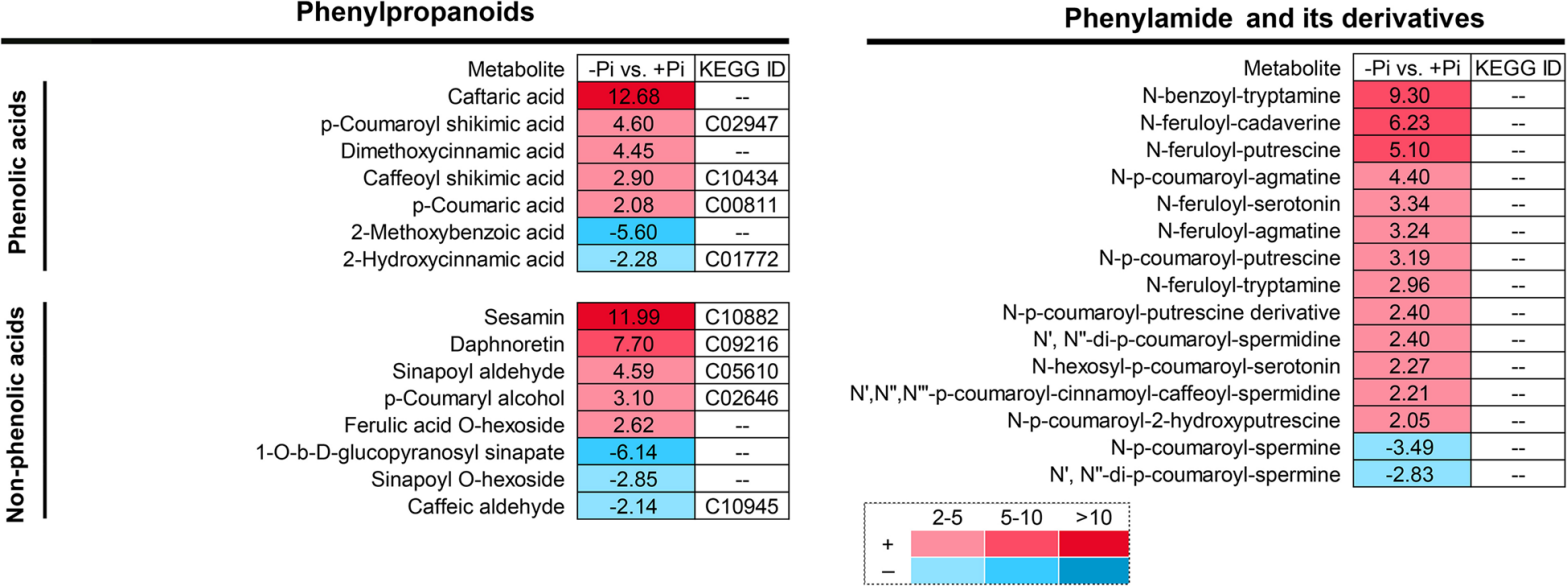
Changes in phenylpropanoids, phenylamides and its derivatives in stylo roots response to low P stress. Numbers indicate the fold changes. Negative numbers represent the inverted fold changes less than 0.5 (−Pi/+Pi < 0.5). KEGG IDs of metabolites are listed in the last column. “--” indicates the metabolite without an ID in KEGG database

**Fig. 10:**
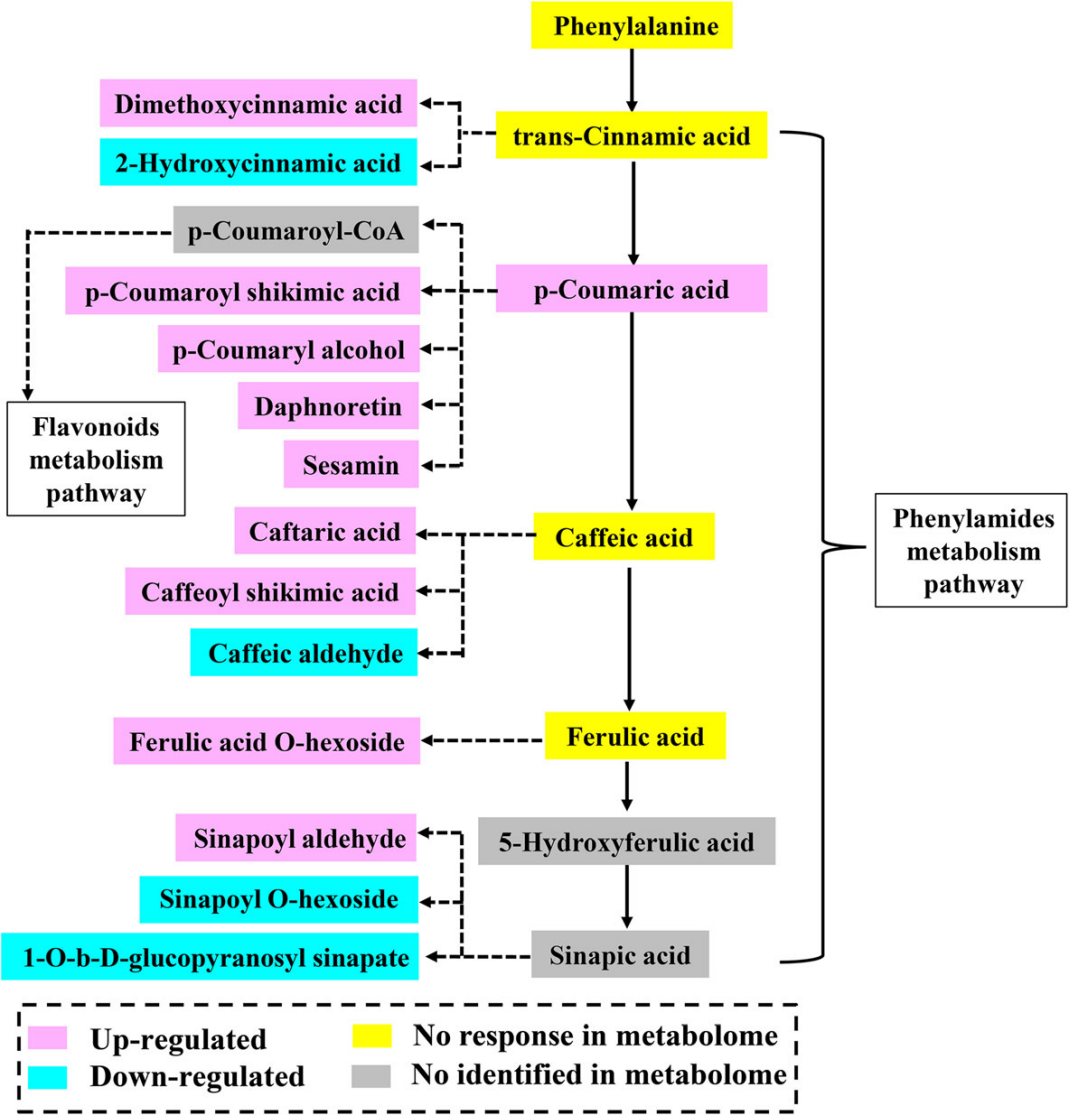
Phenylpropanoids metabolism pathway in stylo roots response to low P stress. The pathway was designed based on KEGG pathway database. The light red, blue, yellow and grey colors indicate up-regulated, down-regulated, no response and no identified metabolites in metabolome, respectively. The solid arrows show direct steps in the pathway, whereas dotted arrows represent indirect and speculated steps in the pathway

In addition, fifteen differentially accumulated phenylamides and its derivatives were identified, including 13 up-regulated and 2 down-regulated DAMs (Fig. 9). The relative levels of 3 phenylamides (N-benzoyl-tryptamine, N-feruloyl-cadaverine and N-feruloyl-putrescine) were increased by more than 5-fold in roots under low P stress. As shown in Fig. 10, numerous metabolites relative to phenylpropanoids metabolic pathway were up-regulated by P deficiency. Interestingly, the concentrations of all of the ρ-coumaric acid and four corresponding derivatives were increased in stylo roots in response to Pi starvation.

## Discussion

### Modification of stylo root morphology in response to low Pi availability

As the major organ for Pi uptake, root is of high plasticity in its developmental response to low Pi availability [52]. Cumulative studies show that the adaptive strategies of plant to P deficiency include changes in root architecture and morphology, such as promotion of lateral root growth, enhancement of root hair development and formation of cluster roots in Proteaceae plants [4]. Such developmental strategies increase the root-soil contact surface, and thus facilitating exploitation of Pi reserves from soils [9]. In this study, root dry weight, root length, root surface area and root volume were significantly increased in stylo under low P treatment for 10 and 15 d, suggesting that stylo adapts to P deficiency through modification of root growth (Fig. 1).

Cell wall loosening is one of important processes during root modification, which can be regulated by expansin genes [33]. Expansins consist of four subfamilies, including α-expansin (EXPA), expansin-like A (EXLA), β-expansin (EXPB) and expansin-like B (EXLB) [53]. To data, three expansin members have been implicated in regulating root architecture and morphology when plants subjected to P deficiency, such as *TaEXPB23* from wheat [54], *GmEXPB2* and *GmEXLB1* from soybean [34, 55]. In this study, the expressions of *SgEXPB2* and *SgEXLB1*, the homologies of *GmEXPB2* and *GmEXLB1*, were increased by Pi starvation in roots (Fig. 2; Additional file 1: Figure S5). Furthermore, the expressions of 4 *SgEXPAs* in stylo roots were also increased under low P stress (Fig. 2; Additional file 1: Figure S5). These results suggest that *expansin* genes play a role in modifications of stylo root growth in responses to P deficiency.

### Accumulation of glucose in P-deficient stylo roots

Plants generally alter the allocation of carbohydrates in response to P deficiency. Large amounts of carbohydrates are allocated to the root system, stimulating root growth and resulting in the increase their root/shoot ratio [56]. In this study, root/shoot ratio in stylo was significantly increased by P deficiency (Fig. 1; Additional file 1: Figure S3). Metabolomic analysis showed that the concentration of glucose increased more than 10-fold in P-deficient stylo roots (Fig. 4). Similarly, accumulations of glucose are observed in tea and ryegrass (*Lolium perenne*) under low Pi condition [43, 57]. Glucose, one of the carbohydrates derived from photosynthesis, is not only involved in plant energy supply, carbon metabolism and cell wall synthesis, but also acted as a crucial signaling molecule [58]. Recently, glucose signaling has been shown to facilitate root growth and development by interacting with phytohormones [59]. In Arabidopsis, it has been demonstrated that glucose crosstalk with auxin signaling displays positive functions in facilitating cell expansion and lateral root formation [59]. These results suggest an important role of glucose in stylo adaptation to P deficiency. Glucose possible function as both energy and signaling molecules for regulating root growth in stylo.

### Scavenging of Pi from P-containing metabolites during P deficiency

Remobilization of P-containing metabolites is considered as an important strategy for plants adaption to P deficiency [15]. Under P-limiting conditions, P-containing metabolites, such as nucleic acid, phospholipids and phosphorylated sugars, can be scavenged to release Pi and maintain P homeostasis in plant cells [60]. It has been shown that phosphorylated sugars are reduced by P deficiency in various plants, such as Arabidopsis [37], barley [40], white lupin (*Lupinus albus*) [61] and *Populus cathayana* [62]. Similarly, the accumulation of 6 phosphorylated sugars were decreased in P-deficient stylo roots (Fig. 4). Furthermore, Pi starvation resulted in decreased concentrations of 2 P-containing cholines and 11 P-containing nucleotides (Fig. 4). Furthermore, a set of genes (4 *SgPAPs* and 3 *SgRNSs*) involved in degradation of P-containing metabolites were up-regulated by Pi starvation in stylo roots (Additional file 1: Figure S7). These results suggest that various changes in P-containing metabolites occur in stylo roots response to Pi starvation.

### P deficiency enhances the levels of flavonoids in stylo roots

Flavonoids, one of the key secondary metabolites, play diverse biological roles in plants, such as transport of auxin, modulation of reactive oxygen species, protection against ultraviolet-B, and interaction with rhizosphere microorganisms [44]. To date, more than 10,000 structural variants of flavonoids have been identified in plants. Based on their basal structures, flavonoids could be divided into flavones, flavanones, flavonols, isoflavones, flavanols, anthocyanins and chalcones [63]. Among them, flavonols (e.g., quercetin, kaempferol, isorhamnetin and kaempferol glycosides) have been found to be accumulated in Arabidopsis during Pi starvation [37]. In this study, the abundances of 41 out of 54 differentially accumulated flavonoids were up-regulated by Pi starvation in stylo roots (Fig. 3b; Fig. 6). The concentrations of kaempferol and its glycoside derivatives belonging to flavonols were significantly enhanced under low Pi condition (Fig. 6). Consistently, S*gFLS-1*, participating in the last step of kaempferol biosynthesis, was up-regulated by Pi starvation in stylo roots (Figs. 7, 8). It has been reported that plant roots can secrete kaempferol, increasing Fe availability by solubilizing Fe complex in soils [44]. Protocatechuic acid, the other flavonoids belonging to flavanols derivatives, plays a similar function with kaempferol in Fe solubilization in rice during Fe deficiency [64]. In this study, increases in the concentrations of protocatechuic acid and its derivative (protocatechuic acid O-hexoside) were observed in stylo roots under low Pi condition (Fig. 6). It is well known that Fe and P can form insoluble complexes in soils. Along with the Fe mobilization by flavonoids, Pi can be released. Therefore, the accumulation of kaempferol and protocatechuic acid in stylo roots might facilitate mobilization of insoluble P.

It has been reported that the levels of isoflavones (e.g., daidzein) are increased in P-deficient common bean (*Phaseolus vulgaris*) [65]. Similarly, the accumulation of daidzein and daizein 7-O-glucoside was enhanced by more than 5-fold in stylo roots under low Pi condition (Fig. 6). Furthermore, a set of genes (*SgUGTs* and *SgHIDs*) involved in daidzein and daizein 7-O-glucoside biosynthesis, were found to be up-regulated by Pi starvation in stylo roots (Figs. 7, 8). It has been demonstrated that daidzein plays an important role in symbiotic network formation with AM fungi, contributing to Pi uptake in soybean roots [63]. In addition, the levels of delphinidin 3-glucoside and peonidin 3-glucoside belonging to anthocyanins, were significantly increased by P deficiency in stylo roots. It has been suggested that the accumulated anthocyanins in plant shoots and leaves could have a photoprotective function, but the functions of anthocyanins in roots remain to be clarified [42].

### Increased accumulation of phenolic acids and phenylamides in P-deficient stylo roots

Except for flavonoids, the other class of secondary metabolites, a set of phenolic acids were also increased in P-deficient stylo roots. For example, the concentration of caftaric acid was enhanced by more than 10-fold under Pi starvation (Fig. 9). It has been reported that phenolic acids exuded from roots can promote mobilization of insoluble P complexes (Fe-P and Al-P), and thus increasing Pi availability [66, 67]. Moreover, caftaric acid is reported to facilitate the interaction of AM fungi with plants [68].

Phenylamides, also referred to hydroxycinnamic acid amides (HCAA) or phenolamides, are synthesized from phenolic acid derivatives and polyamines. It has been reported that phenylamides play important roles in protecting plants against abiotic and biotic stresses [69, 70]. However, the responses of phenylamides to P deficiency are still unclear. In this study, 15 differentially accumulated phenylamides were identified in stylo roots exposed to low P stress (Fig. 9). Among them, the accumulation of 13 phenylamides was increased in P deficient stylo roots, such as N-feruloyl-putrescine (Fig. 9). Recently, N-feruloyl-putrescine has been demonstrated to participate in the interaction with PGPR in rice roots [70]. Therefore, P-deficiency leads to accumulation of flavonoids, phenolic acids and phenylamides, which might facilitate Pi remobilization and cooperate with beneficial microorganisms (e.g., AM fungi and PGPR) in stylo.

## Conclusions

This study demonstrated that stylo root growth were affected by P deficiency, including a wide range of physiological, molecular and metabolic alterations. Under low Pi condition, the enhancement of root growth and *expansin* gene expression probably improve Pi acquisition in stylo. Decreases in P-containing metabolites suggest that internal Pi utilization is enhanced in stylo roots response to P deficiency. Furthermore, the accumulations of flavonoids, phenolic acids and phenylamides were increased in P-deficient stylo roots, which might contribute to plant-environment interactions. Taken together, this study provides a comprehensive understanding of complex responses of stylo roots to Pi starvation.

## Methods

### Plant growth and treatment

In this study, stylo genotype ‘TF0291’ was used, which was originally introduced to Chinese Academy of Tropical Agriculture Sciences (CATAS) from International Center for Tropical Agriculture (CIAT) in Colombia. The seeds of stylo were provided by the Tropical Pasture Research Center, Institute of Tropical Crop Genetic Resources, CATAS, Hainan Province, China.

Stylo seeds were pre-germinated on wet filter paper at 28 °C. After germinated for 3 d, the uniform seedlings were transferred to blue plastic pots containing the modified Magnavaca’s nutrient solution according to Famoso et al. [71]. The nutrient solution contained KCl (1 mmol L^− 1^), CaCl_2_ (1 mmol L^− 1^), Fe-EDTA (77 μmol L^− 1^), MgSO_4_ (200 μmol L^− 1^), Mg (NO_3_)_2_ (500 μmol L^− 1^), NH_4_NO_3_ (1.5 mmol L^− 1^), MnCl_2_·4H_2_O (11.8 μmol L^− 1^), ZnSO_4_·7H_2_O (3.06 μmol L^− 1^), CuSO_4_·5H_2_O (0.8 μmol L^− 1^), H_3_BO_3_ (33 μmol L^− 1^), Na_2_MoO_4_·H_2_O (1.07 μmol L^− 1^), MgCl_2_·6H_2_O (155 μmol L^− 1^), KH_2_PO_4_ (250 μmol L^− 1^). The plants were grown in a greenhouse at Hainan University, China (E121°54′, N30°52′) under a natural day-night cycle and environment. After 7 d of growth, the seedlings were transferred to fresh modified Magnavaca’s nutrient solution supplied with (+Pi) or without (-Pi) 250 μmol/L KH_2_PO_4_. After 0, 7, 10, 15, 20 d of P treatments, shoots and roots were separately harvested for determination of dry weight, total P content, total root length, root surface area and root volume. Roots with different P treatments for 15 d were harvested and stored in − 80 °C for subsequent metabolite and qRT-PCR analysis.

### Assessment of root parameters, total P content, APase activity and other biochemical parameters

To determine root parameters (e.g., root length, surface area and volume), stylo roots were scanned at 400 dpi by a scanner (Epson, Japan). Scanned images were further analyzed through WinRHIZO program (2009, Regent, Canada). After dry weight determination, total P contents in shoots and roots were measured using the phosphorus-molybdate blue color reaction method [72]. A standard curve was used for calculating the P content. APase activities in stylo roots with different P treatments were measured according to Liu et al. [73]. Approximately 0.1 g root samples were ground into powder in 1 mL extraction buffer at 4 °C, then centrifugated at 12,000 g for 10 min at 4 °C, collected the supernatants, incubated in 45 mM sodium acetate buffer containing 1 mM ρ-nitrophenylphosphate at 35 °C for 15 min, measured the production rate of ρ-nitrophenol at absorbance of 405 nm.

The activities of T-AOC and PAL were detected as previously described [74], using the commercial chemical assay kits (Nanjing Jiancheng Bioengineering Institute, Nanjing, China). Briefly, approximately 0.1 g of roots were ground into powder in 0.9 mL extraction buffer of the assay kits at 4 °C. After centrifugation at 12,000 g for 10 min, the supernatants were collected and incubated in reaction solution of assay kits at 37 °C for 30 min, detected the produce rate of F^2+^-TPTZ at absorbance of 520 nm to calculate T-AOC activity. As for PAL activity, the reaction solution was incubated at 30 °C for 30 min to assess the cinnamic acid produce rate by spectrophotometry at absorbance of 290 nm. Protein content in the samples was determined based on the Coomassie Brilliant Blue method [75]. The contents of total phenols and flavonoids were also detected using the commercial chemical assay kits (Jiancheng Bioengineering Institute, Nanjing, China). Approximately 0.1 g root samples were ground into homogenate in 65% ethanol, extracted with ultrasonic extractor at 60 °C for 2 h, then centrifugated at 10,000 g for 10 min and collected the supernatants. After reaction, detected the absorbance at 760 nm and 502 nm to evaluate total phenols and flavonoids contents, respectively. In addition, malate concentration and exudation rate of stylo roots were detected as previously described [76]. Briefly, stylo roots subjected to P treatments for 15 d were transferred to fresh nutrient solution, root exudates and roots were collected respectively. Collected exudate solution was concentrated to 1.2 mL by a freeze-drying vacuum system (Labconco) for determining malate exudation rate. Approximately 0.1 g of stylo roots was used to extract internal malate by grounding with 1.2 mL of 0.25 M HCl, then heated to 80 °C for 20 min, centrifugated at 12,000 g for 15 min, and collecting supernatant for analysis. All samples were analyzed by high performance liquid chromatography (HPLC) method. HPLC (1260 Infinity LC; Agilent 1100) and reversed phase chromatographic column (Kromasil C18, 250 mm × 4.6 mm,5 μm) were applied. The solution (pH 2.8) of 1.56 g NaH_3_PO_4_, 800 mL water, and 16 mL methanol was used as mobile phase. 10 μL sample was added, flow rate was set as 0.8 mL/min, column temperature 25 °C, time 30 min, UV wavelength 214 nm.

### Metabolite analysis

The metabolomic analysis was conducted at MetWare Biotechnology Limited Company (Wuhan, China). All six root samples (three biological repetitions for +Pi and -Pi groups) were used. Metabolites were evaluated through untargeted LC-MS/MS technology. Extraction and metabolite analyses were performed as described by Chen et al. [77]. Briefly, root samples were ground into powder using grinding mill (MM 400, Retsch), 100 mg samples were extracted in 1.0 mL 70% aqueous methanol overnight at 4 °C. Supernatant components were collected and filtered with a 0.22 μm micropore filter (SCAA-104, ANPEL, Shanghai, China) centrifuged at 10,000 g for 10 min before LC-MS/MS analysis.

Two μL of each sample was injected into an Ultra Performance Liquid Chromatography (UPLC, Shim-pack UFLC SHIMADZU CBM30A) equipped with a tandem mass spectrometry (MS/MS, Applied Biosystems 6500 Q TRAP). In UPLC, samples were separated with a reverse-phase Waters Acquity UPLC HSS T3 C18 column (1.8 μm, 2.1 mm × 100 mm). The mobile phase contained eluent A (0.04% acetic acid in aqueous solution) and B (0.04% acetic acid in acetonitrile solution). The gradient of separation program was set at 95:5 (A:B, v/v) at 0 min, 5:95 (A:B, v/v) at 11.0 and 12.0 min, 95:5 (A:B, v/v) at 12.1 and 15.0 min. The flow rate and column temperature were maintained at 0.4 ml/min and 40 °C, respectively. For MS/MS, electrospray ionization (ESI) source operation parameters were set as follows: positive ion mode was used in the instrument; ion source was turbo spray; source temperature was set to 550 °C; ion spray voltage was adjusted to 5.5 kV; the ion source gas I, gas II, curtain gas were set at 55, 60, 25 pounds per square inch, respectively. For triple quadrupole (QQQ) scans, each ion pair was scanned according to the optimized declustering potential and collision energy as multiple reaction monitoring (MRM) experiments.

The qualitative and quantitative analyses of metabolites were conducted according to the previous study [78]. The qualitative analysis of metabolites was performed by matching the secondary spectral information with a self-built MetWare database (MWDB 3.2) and public databases (METLIN: https://metlin.scripps.edu, PubChem: https://pubchem.ncbi.nlm.nih.gov/, mzCloud: https://www.mzcloud.org/). The quantitation of metabolites was analyzed through MRM of QQQ MS/MS. In addition, DAMs were identified by orthogonal projection to latent structure-discriminant analysis (OPLS-DA), according to the criteria of fold change (-Pi/+Pi) more than 2 or less than 0.5, and the variable importance in project (VIP) more than 1.0 [79].

### Quantitative real-time PCR (qRT-PCR) analysis

Total RNA was extracted using the RNA-solve reagent (Omega Biotech, USA). First strand cDNA synthesis from DNase I-treated total RNA was performed using M-MLV reverse transcriptase (Promega, Madison, WI, USA). qRT-PCR was carried out using SYBR Premix Ex Taq II (Takara, Japan) on the Rotor-Gene 3000 qRT-PCR system (Corbett Research, Australia). To reveal the relative levels of candidate genes in response to Pi-starvation, which were related to the modification of plant cell wall, the gradation of P-containing metabolites and synthetic of possible key flavonoid metabolites, based on the ribonucleic acid sequencing (RNA-Seq) data from NCBI database (PRJNA431518, SRX6928121), the expressions of the following genes were detected: *expansin* gene, *SgPAP*, *SgRNS*, *SgF3’H*, *SgFLS*, *SgF3H*, *SgHID*, *SgUGT*. The housekeeping gene, *elongation factor 1-alpha* (*SgEF-1α*, accession number: JX164254), was used as reference gene to normalize gene expression. The primers of genes for qRT-PCR analysis and gene accession numbers were listed in Additional file 2: Table S1. Four biological replicates were included in this study, and the relative expression levels were calculated according to Liu et al. [80]. In addition, multiple sequence alignments and phylogenetic tree of stylo *expansin* family genes were constructed using Clustal X and MEGA 5, respectively.

### Statistical analysis and visualization

The means and standard errors of dry weight, total P content, root parameters, physiological indicators and gene expressions were analyzed by Excel 2016 (Microsoft Corporation, Redmond, WA, USA). One-way analysis of variance (ANOVA) with least significant difference (LSD), Duncan and Student’s *t*-test were performed using SPSS (Version 19.0, IBM, Chicago, IL, USA) software. PCA, volcano diagram, clustered heatmap and circular figures were conducted in program R (v 3.5.1). Before analysis, the raw data was Z score-transformed for normalization in heatmap. The metabolic category figure was created with Microsoft Excel 2016. Metabolic pathways were constructed according to pathway analysis in the Kyoto Encyclopedia of Genes and Genomes (KEGG) metabolic database (http://www.kegg.jp/). To determine the metabolic change folds, untransformed mean values (*n* = 3) were employed to calculate -Pi/+Pi ratios.

## Supplementary information

**Additional file 1: Figure S1** Growth performance of stylo at two P levels. Plants were grown in hydroponics for 15 d with (+Pi) or without (−Pi) 250 μmol/L KH_2_PO_4_ addition. Each bottle contained six plants.** Figure S2** Effects of Pi availability on the increase ratios of root parameters. (a) Total root length. (b) Root surface area. (c) Root volume. After precultured under +Pi (250 μmol/L K_2_HPO_4_) for 7 d, stylo seedlings were transferred into nutrient solution with (+Pi) or without (−Pi) 250 μmol/L KH_2_PO_4_ additions for 7, 10, 15, 20 d. Each bar represents the mean of six replicates with SE (*n* = 6). Different lowercase letters indicate significant difference among groups (*P* < 0.05). Ratio of increase under -Pi (%) = [(−Pi - + Pi)/+Pi] * 100.** Figure S3** Ratio of root/shoot in stylo at two P treatments. Uniform 7 d old stylo seedling was transferred into nutrient solution with (+Pi) or without (−Pi) 250 μmol/L KH_2_PO_4_ additions for 15 d. Asterisks represent significant differences between +Pi and -Pi treatments in the Student’s *t*-test (*: *P* < 0.05, **: 0.001 < *P* < 0.01, ***: *P* < 0.001). **Figure S4** Physiological and biochemical levels in stylo roots response to low P stress. (a) Total P concentration of shoots and roots; (b) APase activity; (c) PAL activity; (d) total phenol content; (e) flavonoid content; (f) T-AOC activity; (g) malate concentration; (h) malate exudation rate. Stylo seedlings were precultured in hydroponics for 7 d with 250 μmol/L KH_2_PO_4_ and subsequently transferred into nutrient solution with (+Pi) or without (−Pi) 250 μmol/L KH_2_PO_4_ additions for 15 d. Each bar represents the mean of four replicates with SE (*n* = 4). Asterisks represent significant differences between +Pi and -Pi treatments in the Student’s *t*-test (*: *P* < 0.05, **: 0.001 < *P* < 0.01, ***: *P* < 0.001). P: phosphate, APase: acid phosphatase, T-AOC: total antioxidant capacity, PAL: phenylalanine ammonia lyase, DW: dry weight, FW: fresh weight. **Figure S5** Phylogenetic analysis of stylo expansin proteins with other expansin proteins in plants. At: *Arabidopsis thaliana*; Gm: *Glycine max*; Ta: *Triticum aestivum*; Sg: *Stylosanthes guianensis*. EXPA: α-expansin; EXLA: expansin-like A; EXPB: β-expansin; EXLB: expansin-like B. **Figure S6** Analysis of stylo root metabolites under two P treatments. (a) Principal component (PC) scores of metabolic the first two variances in stylo roots (*n* = 3). Stylo seedlings were grown in +Pi (250 μmol/L KH_2_PO_4_, triangles) and -Pi (0 μmol/L KH_2_PO_4_, circles) nutrient solutions for 15 d. The confidence level in the grey confidence circle is 95%. (b) Clustered heatmap of differentially accumulated metabolites (DAMs) in stylo roots at low P stress. Individual metabolites are represented by rows and nutritional status are represented by columns. Heatmap visualization of metabolites is based on standardized transformation (Z score) of metabolite concentrations. **Figure S7** Expression analysis of *SgPAPs* and *SgRNSs* genes in response to Pi starvation. Expression levels of five *SgPAPs* and four *SgRNSs* were detected in stylo roots with (+Pi) or without (−Pi) 250 μmol/L KH_2_PO_4_ treatments for 15 d. Each bar represents the mean of four replicates with SE (n = 4). Asterisks indicate significant differences between +Pi and -Pi treatments in the Student’s *t*-test (*: *P* < 0.05, **: 0.001 < *P* < 0.01, ***: *P* < 0.001), ns indicates no significant difference. **Figure S8** Circle graph of seven subcategory flavonoid metabolites in P deficient stylo roots. Digital scale represents the number of corresponding metabolites. Up-regulated or down-regulated metabolites were screened according to the criterion of fold changes ≥2.0 or ≤ 0.5, and VIP ≥1.0 (n = 3).

**Additional file 2: Table S1** A list of primers used for qRT-PCR and gene accession numbers in NCBI database.

**Additional file 3: Table S2** General information of identified metabolites from metabolomic analysis in stylo roots.

**Additional file 4: Table S3** General information about differentially accumulated metabolites (DAMs) from metabolomic analysis in stylo roots.

## Data Availability

The datasets included in this article to support the conclusions and the additional files are also available, the nucleic acid sequence of genes have been submitted to the NCBI database (https://www.ncbi.nlm.nih.gov/nuccore) via BankIt repository with the sequence identifier displayed in Additional file 2: Table S1, and the accession numbers of 38 genes related to this study are as follows: *SgF3’H-1*: MN165117; *SgFLS-1*: MN165118; *SgF3H-1*: MN165119; *SgF3H-2*: MN165120; *SgHID-1*: MN165121; *SgHID-2*: MN165122; *SgHID-3*: MN165123; *SgHID-4*: MN165124; *SgHID-5*: MN165125; *SgUGT-1*: MN165126; *SgUGT-2*: MN165127; *SgUGT-3*: MN165128; *SgEXPA1*: MN540933; *SgEXPA2*: MN540934; *SgEXPA3*: MN540935; *SgEXPA4*: MN540936; *SgEXPA5*: MN540937; *SgEXPA6*: MN540938; *SgEXPA7*: MN540939; *SgEXPA8*: MN540940; *SgEXPA9*: MN540941; *SgEXLA1*: MN540942; *SgEXPB1*: MN540943; *SgEXPB2*: MN540944; *SgEXPB3*: MN540945; *SgEXLB1*: MN540946; *SgEXLB2*: MN540947; *SgEXLB3*: MN540948; *SgPAP1a*: MN540949; *SgPAP1b*: MN540950; *SgPAP10*: KU315545; *SgPAP12*: MN064556; *SgPAP23*: MG492012; *SgRSN1*: MN540951; *SgRSN2*: MN540952; *SgRSN3*: MN540953; *SgRSN4*: MN540954; *SgEF-1a*: JX164254.

## Abbreviations

Al: Aluminum
AM: Arbuscular mycorrhiza
ANOVA: One-way analysis of variance
APase: Acid phosphatase
CATAS: Chinese Academy of Tropical Agriculture Sciences
CIAT: International Center for Tropical Agriculture
Cys: Cystine
DAMs: Differentially accumulated metabolites
EF-1α: Elongation factor 1-alpha
ESI: Electrospray ionization
F3’H: Flavonoid 3′-hydroxylase
F3H: Flavanone 3-hydroxylase
Fe: Iron
FLS: Flavonol synthase
GC-MS: Gas chromatography mass spectrometry
Glu: Glutamate
GPC: Glycerol-3-phosphocholine
HCAA: Hydroxycinnamic acid amides
HID: 2-hydroxyisoflavanone dehydratase
KEGG: Kyoto Encyclopedia of Genes and Genomes
LC-MS: Liquid chromatography mass spectrometry
LSD: Least significant difference
Mn: Manganese
MRM: Multiple reaction monitoring
MWDB: MetWare database
OPLS-DA: Orthogonal projection to latent structure-discriminant analysis
P: Phosphorus
PAE: Phosphorus acquisition efficiency
PAL: Phenylalanine ammonia
PAP: Purple acid phosphatase
PC1: Principal component one
PCA: Principal component analysis
PCho: Phosphocholine
PGPR: Plant growth-promoting rhizobacteria
PHO2: A ubiquitin-conjugating E2 enzyme
PHR1: phosphate starvation response 1
Pi: Soluble inorganic phosphate
PPi: Inorganic pyrophosphate
PSR: Soluble inorganic phosphate starvation response
PUE: Phosphorus utilization efficiency
QQQ: Triple quadrupole
qRT-PCR: Quantitative real-time polymerase chain reaction
RNS: Ribonuclease
SPX: SYG1/PHO81/XPR1
Stylo: Stylosanthes spp.
T-AOC: Total antioxidant capacity
TPTZ: Tripyridine triacridine
UGT: Uridine diphosphate glycosyltransferase
UPLC: Ultra Performance Liquid Chromatography
VIP: Variable importance in project
VPE: Vacuolar Pi efflux transporter
