# Supplementary material for: Metabolic alterations provide insights into Stylosanthes roots responding to phosphorus deficiency
Source: BMC Plant Biol. 2020 Feb 22;20:85. doi: 10.1186/s12870-020-2283-z (PMC7036231; doi:10.1186/s12870-020-2283-z)
Supplement: Supplementary file 1 — Additional file 1: Figure S1 Growth performance of stylo at two P levels. Plants were grown in hydroponics for 15 d with (+Pi) or without (−Pi) 250 μmol/L KH2PO4 addition. Each bottle contained six plants. Figure S2 Effects of Pi availability on the increase ratios of root parameters. (a) Total root length. (b) Root surface area. (c) Root volume. After precultured under +Pi (250 μmol/L K2HPO4) for 7 d, stylo seedlings were transferred into nutrient solution with (+Pi) or without (−Pi) 250 μmol/L KH2PO4 additions for 7, 10, 15, 20 d. Each bar represents the mean of six replicates with SE (n = 6). Different lowercase letters indicate significant difference among groups (P < 0.05). Ratio of increase under -Pi (%) = [(−Pi - + Pi)/+Pi] * 100. Figure S3 Ratio of root/shoot in stylo at two P treatments. Uniform 7 d old stylo seedling was transferred into nutrient solution with (+Pi) or without (−Pi) 250 μmol/L KH2PO4 additions for 15 d. Asterisks represent significant differences between +Pi and -Pi treatments in the Student’s t-test (*: P < 0.05, **: 0.001 < P < 0.01, ***: P < 0.001). Figure S4 Physiological and biochemical levels in stylo roots response to low P stress. (a) Total P concentration of shoots and roots; (b) APase activity; (c) PAL activity; (d) total phenol content; (e) flavonoid content; (f) T-AOC activity; (g) malate concentration; (h) malate exudation rate. Stylo seedlings were precultured in hydroponics for 7 d with 250 μmol/L KH2PO4 and subsequently transferred into nutrient solution with (+Pi) or without (−Pi) 250 μmol/L KH2PO4 additions for 15 d. Each bar represents the mean of four replicates with SE (n = 4). Asterisks represent significant differences between +Pi and -Pi treatments in the Student’s t-test (*: P < 0.05, **: 0.001 < P < 0.01, ***: P < 0.001). P: phosphate, APase: acid phosphatase, T-AOC: total antioxidant capacity, PAL: phenylalanine ammonia lyase, DW: dry weight, FW: fresh weight. Figure S5 Phylogenetic analysis of stylo expansin pro [file 12870_2020_2283_MOESM1_ESM.zip › Additional file 1 Fig. S5.docx]

**Fig. S5**


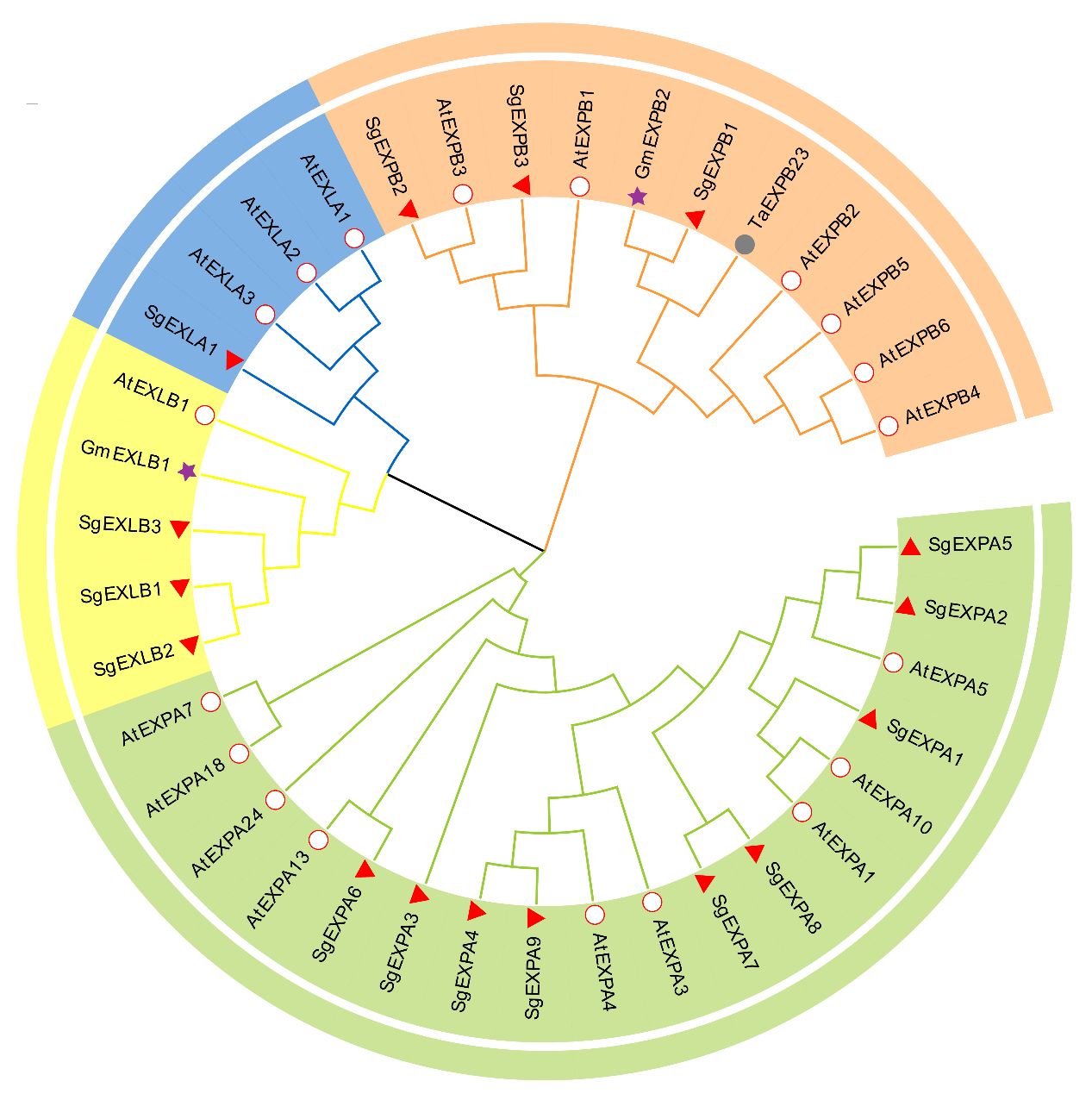


EXPA

EXLA

EXPB

EXLB

**Additional file 1: Fig. S5** Phylogenetic analysis of stylo expansin proteins with other expansin proteins in plants. At: *Arabidopsis thaliana*; Gm: *Glycine max*; Ta: *Triticum aestivum*; Sg: *Stylosanthes guianensis*. EXPA: α-expansin; EXLA: expansin-like A; EXPB: β-expansin; EXLB: expansin-like B.
