# Supplementary material for: Metabolic alterations provide insights into Stylosanthes roots responding to phosphorus deficiency
Source: BMC Plant Biol. 2020 Feb 22;20:85. doi: 10.1186/s12870-020-2283-z (PMC7036231; doi:10.1186/s12870-020-2283-z)
Supplement: Supplementary file 2 — Additional file 2: Table S1 A list of primers used for qRT-PCR and gene accession numbers in NCBI database. [file 12870_2020_2283_MOESM2_ESM.docx]

**Additional file 2: Table S1** A list of primers used for qRT-PCR and gene accession numbers in NCBI database.

| Primer name | Sequence (5’ – 3’) | Gene NCBI accession number |
| --- | --- | --- |
| *SgF3’H-1*-F | CAAGCCCGTAGAATGTGCCTGAT | MN165117 |
| *SgF3’H-1*-R | GGTTCTCCGATTTCTCCGTGTACT |  |
| *SgFLS-1*-F | ATGCCCTGATCTTGTTCTTGGTGT | MN165118 |
| *SgFLS-1*-R | TACGTGCTCTTCTTTGGGTTCTATG |  |
| *SgF3H-1*-F | AGACGGTGGGAAGACATGGATTAC | MN165119 |
| *SgF3H-1*-R | CGGATTCTGGAACGTGGCTATTG |  |
| *SgF3H-2*-F | TATCACTGGCCGGAATCAAC | MN165120 |
| *SgF3H-2*-R | GAGATGGCTAGAGACAATGAAACC |  |
| *SgHID-1*-F | TGGAAGGGCACGAGGAGAGTC | MN165121 |
| *SgHID-1*-R | GGATCGGGGAGTGTGAATGTTA |  |
| *SgHID-2*-F | GGTTGCCGCTCATTATTCCTCC | MN165122 |
| *SgHID-2*-R | AGGGGCCTGGGTAGCGATAGT |  |
| *SgHID-3*-F | CCAACCCCAACAACAACAAACTC | MN165123 |
| *SgHID-3*-R | ATGCGGGTAGTGGTCGGGCT |  |
| *SgHID-4*-F | CCAGACCATTACCAGCCTGCTA | MN165124 |
| *SgHID-4*-R | ATCCTCCGGCCCCCTGTTATC |  |
| *SgHID-5*-F | TTCCTTCACCAACGCTACCTCAACA | MN165125 |
| *SgHID-5*-R | AACGCCGCCACCAACACGAA |  |
| *SgUGT-1*-F | CGACGCCATTGAAGAAAGAGCT | MN165126 |
| *SgUGT-1*-R | ACTCAGTAACCGCCCTCACCA |  |
| *SgUGT-2*-F | GGACTGCAACTGCTGGAAAAAGAG | MN165127 |
| *SgUGT-2*-R | CTGCAAACGCGTCATCTCTAGGT |  |
| *SgUGT-3*-F | GGATCGTTCTTGACTCACTGCG | MN165128 |
| *SgUGT-3*-R | TCAGCCCTCACCCTCTCCTTCT |  |
|  |  |  |
| *SgEXPA1*-F | TGATGCATCAGGGACAATGG | MN540933 |
| *SgEXPA1*-R | TGACTACAATGGAGCCAGGGAG |  |
| *SgEXPA2*-F | CTCTCAACCCATTTTTCAGCACATA | MN540934 |
| *SgEXPA2*-R | TCACATCACCAGCACCTCCAAC |  |
| *SgEXPA3*-F | TTTGCCCTCCTAATTTTGCTCTCC | MN540935 |
| *SgEXPA3*-R | AATCTAACCCCTCCATGCTTCTTG |  |
| *SgEXPA4*-F | AGTGCGCAAATGACAAGGAGTG | MN540936 |
| *SgEXPA4*-R | GTGAACCTAATCCCACCGTGCT |  |
| *SgEXPA5*-F | TCATCCCCTGCATCCTCCATT | MN540937 |
| *SgEXPA5*-R | AACCTTATTCCCCCTCTTCTCTTG |  |
| *SgEXPA6*-F | TCCGATTGAGGCATTTGAGAAGAT | MN540938 |
| *SgEXPA6*-R | ACGCGTTTATGTGCCAATTCTG |  |
| *SgEXPA7*-F | CACTATGGGAGGTGCTTGTGGA | MN540939 |
| *SgEXPA7*-R | CAAAGTGCTGCAAAGGAGGGT |  |
| *SgEXPA8*-F | TGGCTTGAGCTGTGGGTCTTG | MN540940 |
| *SgEXPA8*-R | GGTTCGGCGAGATCAAAGTGTT |  |
| *SgEXPA9*-F | CGAGTGACAATGGTGGATGGTGT | MN540941 |
| *SgEXPA9*-R | GAACCCTTCACGTACGTCCTCACT |  |
| *SgEXLA1*-F | CGATCGCTGTCTCCACCAATC | MN540942 |
| *SgEXLA1*-R | GCTCTGCTGCTAAGAACAAAATCAG |  |
| *SgEXPB1*-F | GCTGCCGTAATTGAATATGAGGATG | MN540943 |
| *SgEXPB1*-R | GAAAGGTGCTCTGAGTGGTGATCC |  |
| *SgEXPB2*-F | TGTGCGGCGGAGATACAACCT | MN540944 |
| *SgEXPB2*-R | GACCCACTGCACCCACCCTT |  |
| *SgEXPB3*-F | TGTTCCAAGCGAGCGGTGAC | MN540945 |
| *SgEXPB3*-R | CACGGAAGGCAATGTTTTTACC |  |
| *SgEXLB1*-F | CGCATGCTATCAGATTCGGTGTA | MN540946 |
| *SgEXLB1*-R | CATGGAGCTTAAGAAGGACATTGG |  |
| *SgEXLB2*-F | CGCAGCTACAACCCACACTACTTAG | MN540947 |
| *SgEXLB2*-R | TACACCCCTGCACTTCCACTCACT |  |
| *SgEXLB3*-F | TGTGCAAGGCGAATGGAGTAAC | MN540948 |
| *SgEXLB3*-R | AATGTTGCGGGGGAAGTAGTTG |  |
|  |  |  |
| *SgPAP1a*-F | GCGCGGCTCAATCAACACTAC | MN540949 |
| *SgPAP1a*-R | TGCCAAAACACCACATTCTCCTC |  |
| *SgPAP1b*-F | GTACCTGCTGAGAACCGTGAAAAAT | MN540950 |
| *SgPAP1b*-R | CCGGCCGAAGAATAACCAAGTA |  |
| *SgPAP10*-F | GCGAGAACAGCAAGCAAAAGAAG | KU315545 |
| *SgPAP10*-R | GTGTGTGTGATTCCGACTTCATAGT |  |
| *SgPAP12*-F | TTCAGAACCCCACCCCCTCTT | MN064556 |
| *SgPAP12*-R | GAACCTTCCCCAAGTATCCCATCT |  |
| *SgPAP23*-F | CAAAGAAGATGCAGTTGGTGACC | MG492012 |
| *SgPAP23*-R | CTGATAGAGTCGGCCGGAAG |  |
|  |  |  |
| *SgRSN1*-F | TCCGATCGAGCCTTTCTTCTCC | MN540951 |
| *SgRSN1*-R | TGATACCGTTGCACATAGTTCCCG |  |
| *SgRSN2*-F | CATGGAACCTGTGCTGAATCTGAG | MN540952 |
| *SgRSN2*-R | CTTGGTAGAGTTGGCTGTTATGTGC |  |
| *SgRSN3*-F | ACAGGCCTGCTTCACACAATGC | MN540953 |
| *SgRSN3*-R | TGTCCTTCAAGTCGAGACCTGCT |  |
| *SgRSN4*-F | GCAGAGGGCCAACATCCTAAAAG | MN540954 |
| *SgRSN4*-R | GGCACTGGATGAAGGCTGAAG |  |
|  |  |  |
| *SgEF-1a*-F | CACTTCAGGACGTGTACAAGATC | JX164254 |
| *SgEF-1a*-R | CTTGGAGAGCTTCATGGTGCA |  |
